# Accumulation of uPA – PAI-1 complexes inside the tumour cells is associated with axillary nodal invasion in progesterone-receptor-positive early breast cancer

**DOI:** 10.1038/sj.bjc.6600656

**Published:** 2003-01-28

**Authors:** J Schneider, M Pollán, A Tejerina, J Sánchez, A R Lucas

**Affiliations:** 1 Fundación Tejerina-Centro de Patología de la Mama, Madrid, Spain; 2 Facultad de Ciencias de la Salud, Universidad Rey Juan Carlos, Madrid, Spain; 3 Departamento de Epidemiología del Cáncer, Centro Nacional de Epidemiología, Madrid, Spain; 4 Universidad de Alcalá, Madrid, Spain

**Keywords:** early breast cancer, uPA, PAI-1, axillary nodes, metastasis

## Abstract

Both urokinase-like plasminogen activator (uPA) and its inhibitor plasminogen activator inhibitor (PAI-1), as well as uPA–PAI-1 complexes, have been identified as important prognostic factors in breast cancer. We have recently reported that the latter are identifiable inside breast cancer cells by means of immunohistochemistry. Using this technique, we have studied a series of 212 early (pT1) unifocal breast cancers and have correlated the expression of uPA–PAI-1 complexes, together with other clinical and biological features (histologic variety, histologic and nuclear grade, hormone receptors, Ki67 labelling index, c-erb-B2-, p53- and CD44std-expression) with or without the occurrence of axillary node invasion. In a logistic regression model, looking for associations with axillary metastasis, we found a statistically significant interaction between the presence of uPA–PAI-1 complexes and progesterone receptor positivity (*P*=0.04). A final model showed that the presence of uPA–PAI-1 complexes was a determinant factor for axillary metastasis among women carrying tumours expressing progesterone receptors. In these cases, the presence of uPA–PAI-1 complexes carried with it a nearly 14-fold risk of axillary node invasion (*P*=0.009). These results may indicate that small, hormone-receptor-positive breast cancers (with a theoretical good prognosis) may carry an elevated risk of nodal involvement if accumulation of uPA–PAI-1 complexes is shown inside their tumour cells by means of immunohistochemistry.

Among the mechanisms involved in tumour invasion and metastasis, basement membrane and, more generally, extracellular matrix degradation is likely to play a major role. Several physiologically active enzymes are used for this purpose by the tumour cells. The plasminogen activation system, necessary for clot degradation, and also involved in wound healing and inflammation, is one of such mechanisms incorporated by tumour cells for developing the metastatic phenotype (Andreasen *et al*, 1997). Two natural plasminogen activators are known, tissue-type plasminogen activator (tPA) and urokinase-like plasminogen activator (uPA). Of them, it is the latter, uPA, which plays the most important role in basement membrane, and, more generally, peritumoral matrix degradation. The inactive form of uPA (pro-uPA) is secreted mainly by stromal cells (and to a much lesser extent also by tumour cells), and is activated by binding to its cell-surface receptor (uPAR). Active receptor-bound uPA in its turn activates the passage of plasminogen to plasmin, a strong proteolytic enzyme, which furthermore activates metalloproteinases, thus creating a localised microenvironment of matrix degradation, through which migration of tumour cells is facilitated. In the long run, however, such an unspecific proteolytic effect in the immediate surrounding of the tumour cell would be dangerous for the tumour cell itself, so that the whole process is held in control by a negative feedback loop, mediated by specific inhibitors of uPA, called plasminogen activator inhibitors (PAI). Two of them are known (PAI-1 and PAI-2), although it seems that, in practice, it is mainly PAI-1 that comes into play in solid tumours. Only the active form of uPAR-bound uPA interacts with PAI-1, forming stable uPA–PAI complexes that are internalised (Olson *et al*, 1992). We have recently been able to visualise this step of the chain by means of immunohistochemistry, using an antibody that selectively reacts with uPA–PAI-1 complexes (Schneider *et al*, 2000). In the same study, we also found that hormones seem to play a role in the whole process, because the accumulation of uPA–PAI complexes inside the tumour cells was significantly associated with the expression of oestrogen and progesterone receptors by them.

Both uPA and its inhibitor PAI-1, as well as uPA–PAI-1 complexes, have been identified as important prognostic factors in breast cancer. Schmitt *et al* (1991) were the first to report that PAI-1 expression is associated with a significantly worse outcome in breast cancer, and these initial findings were corroborated by the same group later, after long-term follow-up (Harbeck *et al*, 1999), as well as by Foekens *et al* (2000). Similar results were obtained for uPA by Grøndahl-Hansen *et al* (1993). Jänicke *et al* (2001) found the combination of uPA/PAI-1 to have a negative prognostic impact on breast cancer. The variable fraction of stable uPA–PAI complexes, finally, which is formed as a result of the interaction between uPA and its inhibitor PAI-1, has been associated with a better prognosis by Pedersen *et al* (2000), but also with a worse prognosis by Sten-Lindner *et al* (2001).

Taking into consideration the decisive role of the uPA–PAI system in the metastatic process, we have carried out the present study in order to verify if the presence of uPA–PAI-1 complexes, together with other biological features of the primary tumour, aids in predicting axillary nodal metastasis in early breast cancer at the time of surgery.

## Materials and Methods

For this study, only unifocal invasive tumours with a pathologically verified diameter of 2 cm or less (pT1) were considered. Between January 1993 and December 2000, 212 patients harbouring tumours showing these features were operated upon at our institution. Of them, the vast majority were infiltrating ductal carcinomas (178; 84%), followed by lobular infiltrating carcinomas (16; 7.5%), pure tubular carcinomas (10; 4.7%) and other, less frequent histologic varieties (8; 3.8%). Substaging according to size was as follows: pT1a (0.1–0.5 cm); nine cases (4.2%); pT1b (0.6–1.0 cm); 51 cases (24.1%); pT1c (1.1–2.0 cm), 152 cases (71.7%). A complete axillary dissection was carried out in every patient. More than 10 axillary nodes were removed in all of them (range: 18–52). Axillary metastasis was present in 54 out of the 212 patients of the study (25.5%). None of the patients with pT1a tumours had invaded nodes *vs* 9/51 of those carrying pT1b tumours (17.6%), and 45/152 (29.6%) of patients with pT1c tumours.

Besides histology and tumour size, the other variables of the study taken into consideration for predicting axillary node invasion were as follows: histologic and nuclear grade (33 unknown), oestrogen and progesterone receptor expression (three unknown), the Ki67 labelling index (three unknown), c-erb-B2 expression (26 unknown), mutant p53 expression (23 unknown), CD44std expression (30 unknown), and finally uPA –PAI-1 complex accumulation inside the tumour cells (23 unknown).

### Immunohistochemistry

**Figure 1:**
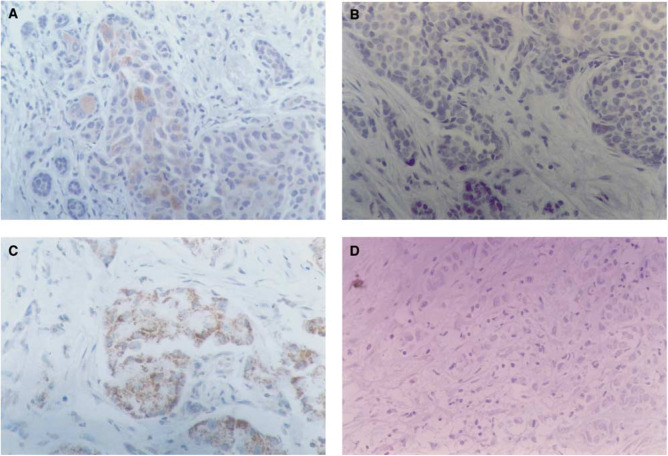
Immunohistochemical detection of uPA-PAI-1 complexes in breast cancer. Streptavidin-biotin peroxidase X 250. (**A**) Strong reaction in <10% tumour cells. Very weak reaction in the rest of tumour cells. No reaction in stromal cells. (**B**) Negative control of **A**). (**C**) Strong, granular reaction in most tumour cells. No stromal reaction. (**D**) Negative control of **C**).

The immunohistochemical technique employed has been extensively described elsewhere (Schneider *et al*, 1999,^2000^). Briefly, 5-*μ*m paraffin sections were mounted on poly L-lysine-coated slides for heat-induced epitope retrieval (‘HIER’ technique) in citrate buffer. We used the same, commercially available streptavidin – biotin – peroxidase kit (Histostain-SP, Zymed, San Francisco, CA, USA) throughout the whole procedure, to ensure uniformity of results. The antibodies employed were as follows: NCL-CB11 (c-erb-B2), NCL-ER-6F11 (ER), NCL-p53-D07 (p53), all from Novocastra Laboratories, Newcastle, UK; prediluted MIB1 (Ki67) and PR-2C5 (PR) from Zymed, San Francisco, CA, USA; BMS113 (CD44std) from Boehringer Ingelheim, Germany; AB775 (uPA–PAI-1 complexes) from Chemicon International, Temecula, CA, USA (which is the same as the former antibody BMS4139 from Boehringer Ingelheim, Germany). This antibody was developed and characterised by Cederholm-Williams *et al* (1985) and shown by reverse zymography to selectively recognise uPA–PAI-1 complexes. We recently demonstrated by means of immuno-histochemistry that it reacts with uPA–PAI-1 complexes (Schneider *et al*, 2000), and that the reaction is located intracellularly, as predicted by means of biochemical methods by Olson *et al* (1992). Negative controls were carried out in parallel by omitting the first antibody (Figure 1

). The incubation time was 1 h at room temperature in a humid chamber for all antibodies, which, apart from the prediluted MIB1-Ki67 solution, which was directly used as supplied, were employed at following dilutions: NCL-CB11 (c-erb-B2), 1 : 40; NCL-ER-6F11 (ER), 1 : 100; NCL-p53-D07, 1 : 100; BMS113 (CD44std), 1 : 100; AB775 (uPA–PAI-1 complexes), 1 : 1.000. The evaluation of nuclear staining patterns (ER, PR, Ki67 and p53) was straightforward, since tumours positive for ER, PR or p53 always showed specific staining in more than 10% of tumour cells. The Ki67 labelling index was expressed as the percentage of reactive tumour cells. For the evaluation of CD44std and uPA–PAI-1 staining, finally, we adopted the scoring system used by us in the past (Schneider *et al*, 1999,^2000^), which takes into account both the staining intensity and the proportion of reactive tumour cells, and gives a final score ranging between 0 and 6 (6 corresponding to the highest expressors, showing intense staining in more than 20% of the tumour cells). The slides were evaluated twice in a blinded fashion, with an interval of 1 month, after reshuffling. In those cases where a discrepancy arose between the first and the second evaluation (less than 10% of the cases), a consensus was reached.

### Statistics

The strength of the association between uPA–PAI-1 complex expression and the rest of variables with the presence of axillary node involvement was assessed computing the corresponding odds ratios using unconditional logistic regression. In a second step, the possible interaction between uPA–PAI-1 complexes and other variables was explored through a stratified analysis and the statistical significance was computed via logistic regression including the corresponding interaction term. Given the different results obtained according to the presence or absence of progesterone receptors, and that the effect of uPA–PAI-1 complexes seemed to be restricted to tumours with progesterone receptors, the multivariate model was fitted taking this interaction into account. Furthermore, the multivariate analysis was also built in independently in women with and without progesterone receptors. The statistical analysis was carried out using the STATA statistical package (Stata Corporation, TX, USA).

## Results

### Univariate and bivariate analysis

All clinical and biological tumour variables potentially associated with axillary node invasion were individually tested in a univariate model (Table 1

**Table 1:** Prognostic factors related to axillary lymph node invasion in early (T1) breast cancer: Univariate analysis

	**Number of women**	**No. of women with positive nodes (%)**	**OR**	**95% CI**	***P*-value**
					
*Histologic type*					
Ductal	178	47 (26%)	1		
Lobular	16	5 (31%)	1.26	0.42–3.84	0.676
Tubular	10	0 (0%)	0.00	0.00–1.09	0.069
Rest of types	8	2 (25%)	0.93	0.18–4.76	0.930
					
*Tumour size*					
>1 cm	152	45 (30%)	1		
1–0.6 cm	51	9 (18%)	0.51	0.23–1.13	0.098
⩽0.5 cm	9	0 (0%)	0.00	0.00–1.04	0.063
					
*uPA–PAI-1*					
0	55	10 (18%)	1		
1–4	48	15 (31%)	2.04	0.82–5.12	0.126
5–6	86	25 (29%)	1.84	0.81–4.22	0.148
Unknown	23	4 (17%)	0.80	0.26–3.40	0.934
					
*Hormone receptors*					
ER−PR−	61	14 (23%)	1		
ER+PR−	24	7 (29%)	1.38	0.48–4.00	0.551
ER+PR+	124	33 (27%)	1.21	0.59–2.49	0.591
Unknown	3	0 (0%)	0.00	0.00–4.68	1.000
					
*Nuclear grade*					
1 and 2	122	32 (26%)	1		
3	57	16 (28%)	1.10	0.54–2.22	0.796
Unknown	33	6 (18%)	0.63	0.24–1.65	0.343
					
*Histologic grade*					
1 and 2	139	31 (22%)	1		
3	40	17 (43%)	2.58	1.22–5.41	0.013
Unknown	33	6 (18%)	0.77	0.29–2.04	0.605
					
*Ki67*					
⩽10%	89	16 (18%)	1		
>10%	99	35 (35%)	2.50	1.26–4.93	0.008
Unknown	24	3 (13%)	0.65	0.17–2.45	0.527
					
*c-erb-B2*					
Negative	159	43 (27%)	1		
Positive	27	7 (26%)	0.94	0.37–2.39	0.904
Unknown	26	4 (15%)	0.49	0.16–1.51	0.213
					
*p53*					
Negative	154	41 (27%)	1		
Positive	35	10 (29%)	1.10	0.49–2.49	0.815
Unknown	23	3 (13%)	0.41	0.13–1.46	0.171
					
*CD44*					
3–6	110	18 (16%)	1		
1–2	22	5 (23%)	1.50	0.49–4.60	0.475
Negative	50	25 (50%)	5.11	2.41–10.8	<0.001
Unknown	30	6 (20%)	1.28	0.45–3.57	0.640

). Only histologic grade (*P*=0.013), high proliferation (Ki67>10%; *P*=0.008) and absence of CD44std expression (*P*<0.001) emerged as significant predictors of axillary metastasis, in agreement with the results previously published by us (Schneider *et al*, 1999). In fact, the present series of early breast cancers is an extension of that previously published one, and these results are now confirmatory of the ones obtained in that preliminary study. Any degree of uPA–PAI-1 complex accumulation inside the tumour cells showed a trend towards a significant association with nodal metastasis. Thus, in the further analyses carried out, tumours were termed uPA–PAI-1-positive or –-negative, regardless of the number of staining cells, or the intensity of the reaction. Small tumour size and tubular histology, conversely, showed a trend towards statistically significant absence of invaded nodes (see Table 2

**Table 2:** Stratified analysis of uPA–PAI categories by the rest of the predictors. Statistical significance of the interaction between uPA–PAI complexes and the other variables

	**Number of women**	**Women with positive nodes (%)**	**OR^a^**	**95% CI^a^**	***P*-value of the estimator^a^**	***P*-value of the interaction^b^**
**Histologic type**						
*Ductal*						
uPA negative	39	7 (18%)	1.00			
uPA positive	122	38 (31%)	2.07	0.84–5.10	0.115	
*Other*						
uPA negative	16	3 (19%)	1.00			
uPA positive	12	2 (17%)	0.87	0.12–6.21	0.887	0.419
						
**Tumour size**						
>1 cm						
uPA negative	43	9 (21%)	1.00			
uPA positive	93	32 (34%)	1.98	0.85–4.64	0.115	
⩽1 cm						
uPA negative	12	1 (8%)	1.00			
uPA positive	41	8 (20%)	2.67	0.30–23.8	0.380	0.804
						
**Receptors**						
*ER−PR−*						
uPA negative	22	6 (27%)	1.00			
uPA positive	36	8 (22%)	0.76	0.22–2.59	0.663	
*ER+PR−*						
uPA negative	3	1 (33%)	1.00			
uPA positive	16	4 (25%)	0.67	0.05–9.47	0.765	0.929
*ER+PR+*						
uPA negative	30	3 (10%)	1.00			
uPA positive	80	28 (35%)	4.85	1.35–17.4	0.016	0.040
						
**Nuclear grade**						
*1–2*						
uPA negative	20	2 (10%)	1.00			
uPA positive	88	28 (32%)	4.20	0.91–19.4	0.066	
*3*						
uPA negative	19	5 (26%)	1.00			
uPA positive	35	11 (31%)	1.28	0.37–4.46	0.695	0.239
						
**Histologic grade**						
*1–2*						
uPA negative	31	3 (10%)	1.00			
uPA positive	94	26 (28%)	3.57	1.00–12.8	0.050	
*3*						
uPA negative	8	4 (50%)	1.00			
uPA positive	29	13 (45%)	0.81	0.17–3.89	0.795	0.151
						
*Ki67*						
⩽10%						
uPA negative	25	3 (12%)	1.00			
uPA positive	52	10 (19%)	1.75	0.44–7.01	0.432	
*>10%*						
uPA negative	25	7 (28%)	1.00			
uPA positive	66	27 (41%)	1.78	0.65–4.85	0.259	0.982
						
**c-erb-2**						
*Negative*						
uPA negative	42	8 (19%)	1.00			
uPA positive	97	31 (32%)	2.00	0.83–4.82	0.124	
*Positive*						
uPA negative	7	2 (29%)	1.00			
uPA positive	20	5 (25%)	0.83	0.12–5.72	0.853	0.419
						
**p53**						
*Negative*						
uPA negative	41	8 (20%)	1.00	0.78–4.62		
uPA positive	92	29 (32%)	1.90	0.157		
*Positive*						
uPA negative	8	2 (25%)	1.00			
uPA positive	27	8 (30%)	1.26	0.21–7.65	0.799	0.691
						
**CD44**						
*Positive*						
uPA negative	36	4 (11%)	1.00	0.49–4.60	0.242	
uPA positive	90	18 (20%)	2.00			
*Negative*						
uPA negative	18	6 (33%)	1.00			
uPA positive	32	19 (59%)	2.92	0.87–9.78	0.082	0.657

a Estimators and *P*-values obtained from independent logistic models.

b *P-*value obtained from a single logistic model including the interaction term.

).

Table 3

**Table 3:** Prognostic factors related to axillary node invasion in early (T1) breast cancer: multivariate analysis including the interaction between uPA–PAI and positive progesterone receptors (PR+)

	**OR**	**95% CI**	***P*-value**
			
*Tumour size*			
>1 cm	1		
⩽1 cm	0.34	0.12–1.00	0.051
			
*Hormone receptors and uPA–PAI*^*a*^			
ER− and PR−	1	0.20–4.66	0.955
ER+ and PR−	0.96	0.04–2.29	0.199
ER+ and PR+, and uPA–PAI negative^a^	0.31	1.28–11.6	0.017
ER+ and PR+, and uPA–PAI positive^a^	3.85		
			
*Histologic grade*			
1–2	1		
3	2.56	0.90–7.27	0.077
			
*Ki67*			
⩽10%	1		
>10%	2.09	0.76–5.75	0.154
			
*CD44*			
Positive	1		
Negative	6.86	2.69–17.5	<0.001

a The interaction term PR and uPA–PAI was statistically significant (*P*=0.032), but uPA–PAI complexes only showed any effect in PR+ tumours. The model was therefore adapted to consider this variable only among PR+ cases (see text).

depicts the stratified analysis of the effect of uPA–PAI-1 complexes by categories of the other variables. The statistical significance of the interaction between uPA–PAI and that particular variable is presented in the last column. The most interesting finding in this analysis was the interaction observed between uPA–PAI-1 and oestrogen and progesterone receptor positivity (ER+PR+). The prognostic effect of uPA–PAI-1 complexes seemed to be restricted to those cases with hormonal receptors. All tumours expressing progesterone receptors did also express oestrogen receptors. The contrary was however not always the case. When testing these oestrogen-receptor-positive, progesterone-receptor-negative tumours (ER+PR−) in association with uPA–PAI-1 complexes, there appears no increased incidence of nodal invasion. Thus, in the above-mentioned case of tumours with both oestrogen and progesterone receptor expression, the significant increase of nodal metastasis related to uPA–PAI-1 complexes seems to be associated with progesterone-receptor positivity only. While the number of ER+PR− tumours giving rise to this hypothesis was comparatively small (19 out of 189), the OR associated with uPA–PAI-1 was remarkably similar to the one observed among patients without hormonal receptors and neither of them was close to being statistically significant.

### Multivariate analysis

According to the above-mentioned results, the subsequent multivariate analysis was carried out including an interaction term between PR and uPA–PAI-1 complexes. While this term was statistically significant (*P*=0.032), the main effect for uPA–PAI complexes was very close to 1 (OR=0.98). Thus, we readapted the model considering the uPA–PAI-1 effect only inside the group of PR-positive tumours. These results are presented in Table 3.

The final multivariate model (Table 3) confirmed the independent prognostic strength of CD44 and suggests an important role for tumour size and histologic grade, even though these two variables failed to attain statistical significance. The protective role for hormonal receptors seemed to be restricted to the presence of both ER and PR. However, uPA–PAI-1 complexes reversed this protective effect, producing an almost four-fold increase of risk.

Finally, when the multivariate model was applied maintaining the stratification by progesterone receptor expression (Table 4

**Table 4:** Prognostic factors related to axillary lymph node invasion in early (T1) breast cancer: multivariate analysis stratified by progesterone receptor expression

	**Tumours without progesterone receptors**			**Tumours with progesterone receptors**		
	**OR**	**95% CI**	***P*-value**	**OR**	**95% CI**	***P*-value**
						
*Tumour size*						
>1 cm	1			1		
⩽1 cm	0.53	0.07–3.74	0.522	0.27	0.07–1.02	0.053
						
*uPA–PAI-1*						
Negative	1			1		
Positive	1.24	0.26–6.00	0.790	13.79	1.95–97.4	0.009
						
*ER*						
ER−	1					
ER+	0.55	0.09–3.27	0.513	—	—	—
						
*Histologic grade*						
1–2	1			1		
3	0.75	0.14–3.96	0.737	6.68	1.39–32.1	0.018
						
*Ki67*						
⩽10%	1			1		
>10%	7.68	0.68–3.96	0.099	1.48	0.45–4.89	0.517
						
*CD44*						
Positive	1			1		
Negative	13.37	2.89–61.7	0.001	4.50	1.28–15.9	0.019

), the prognostic value for predicting axillary invasion of tumour size, histologic grade, CD44std-negativity and uPA–PAI-1 complex accumulation was only observed in progesterone-receptor-positive tumours, whereas for their progesterone-receptor-negative counterparts, the only independent predictor was CD44std. In this group, a Ki67-labelling index >10% was also associated with an almost significantly increased risk.

## Discussion

Pedersen *et al* (2000) have studied uPA–PAI-1 complex levels in breast cancer patients using a different method from the one employed by us. Their results and ours are also not strictly comparable in that we studied only early-stage unifocal cancers of less than 2 cm diameter, whereas they included into their study all stages of the disease. Pedersen *et al* measured the uPA–PAI-1 fraction biochemically in tumour tissue extracts, and correlated the obtained results both with the incidence of nodal inavsion and with survival. They found that a high uPA–PAI-1 complex fraction was associated with a longer survival and a lower incidence of axillary nodal invasion. However, these relations lost statistical significance in their multivariate analysis, in favour of total PAI-1 levels and lymph node status. The apparent discrepancy between our results and those reported by Pedersen *et al* may be attributable to the difference in the tumours studied: whereas, as has been said, we restricted our analysis to early, T1 breast cancers, they included all stages into their analysis. As can be seen from their univariate analysis, they found a highly significant (*P*<0.0001) association between uPA–PAI-1 complexes and a tumour size of 2 cm or less. This may in itself explain the significant association found in their univariate analysis with a better survival and lower incidence of axillary metastasis (it may also explain, incidentally, why the latter associations are lost in the multivariate analysis). Even within our very selected group of small tumours, we also found a trend towards an association of uPA–PAI-1 complex expression and lower tumour size. In fact, uPA–PAI-1 complexes were detected by us in 41 out of 53 (77.4%) T1a, b tumours, with a diameter of 1 cm or less *vs* 93 out of 136 (68.3%) T1c tumours, with a diameter between 1.1 and 2 cm. However, this difference did not attain statistical significance (*P*=0.29). In opposition to the findings of Pedersen *et al* Sten-Lindner *et al* (2001) have recently reported a significantly worse relapse-free survival for patients with breast cancers showing higher cytosol concentrations of uPA–PAI-1 complex. Our results are more in agreement with these latter ones, since axillary nodal invasion is directly related to survival.

Interestingly, we found in our series of early-stage breast cancers that the association of uPA–PAI-1 complex and progesterone receptor expression predicted a significantly higher incidence of axillary node metastasis. This relation was maintained throughout the whole statistical analysis, including the multivariate model, where the odds ratio for axillary node invasion in tumours expressing both uPA–PAI-1-complexes and progesterone receptors was 13.79. Although the 95% confidence interval for this odds ratio is very large (1.95–97.4), it is placed far to the right of the null line, and thus statistically significant, in contrast with the results obtained for the group of tumours not expressing progesterone receptors (Table 4). Although the statistical evidence is solid, our results must still be interpreted with a certain degree of caution. In fact, unlike clinical drug trials, where protocols prespecify which analyses should be carried out, and which should be considered primary and secondary, a study such as ours, where the prognostic power of a given biological marker is investigated for the first time, is much less rigid in this sense. A number of interactions are tested in a logistic regression model, and if an interesting one is found, it is followed through to the end, such as we have done. In this context, there is still a small chance for serendipity, which must always be considered. Nevertheless, and with these limitations in mind, we still believe that the results are solid, since the interaction of progesterone receptor- and uPA–PAI-complex-positivity has been tested from every possible angle, and, as Table 4 clearly shows, there seem to exist huge biological differences in relation to the expression or not of progesterone receptors by the tumours, especially in association with the expression of uPA–PAI complexes by them.

Hormone receptor expression is generally considered a biological feature of good prognosis in breast cancer. Why progesterone receptor expression, when associated with uPA–PAI-1 complex expression, might favour axillary metastasis is therefore puzzling at first sight. However, recent studies performed on endometrial stromal cells have shown that epithelial growth factor (EGF) expression and progesterone receptor expression are both necessary for maximal PAI-1 expression (Lockwood, 2001). PAI-1, besides uPA inactivation, has been shown to have other paradoxical roles, which may explain why PAI-1 levels are one of the most powerful ominous prognostic factors in breast cancer. So, Chazaud *et al* (2002) have shown PAI-1 to induce the formation of filopodia and to facilitate the migration of breast cancer cells, suggesting a chemokine-like, metastasis-enhancing role for PAI-1. Furthermore, they found that, in order to exert this promigratory effect, the uPAR–uPA–PAI-1 system must be functioning in all its parts, including the last step, which involves the internalisation of uPA–PAI-1 complexes by means of endocytosis, mediated by the low-density lipoprotein receptor-related protein (LRP). In our study, we have detected the final link of the chain, that is, the presence of uPA–PAI-1 complexes inside the tumour cell. According to the just cited study by Chazaud *et al*, this implies that all previous steps have taken place, including PAI-1 expression, which, according to Lockwood *et al*, is directly related to progesterone receptor expression. Pedersen *et al* (2000) and Sten-Lindner *et al* (2001), finally, have convincingly shown that the uPA–PAI-1-complex fraction is directly related to the amount of total PAI-1.

All these findings taken together seem to indicate that our tumours expressing both uPA–PAI-1 complexes and progesterone receptors are probably the ones also expressing the highest levels of PAI-1, with its inherent bad prognosis, and also showing the highest activity of the uPAR–uPA–PAI-1 system, with its promigratory, metastasis-enhancing effect on breast cancer cells.

In conclusion, the detection of uPA–PAI-1 complexes inside the tumour cells in early (T1) breast cancers expressing progesterone receptors is associated with a significantly higher incidence of axillary metastasis, and this might be of clinical value at the present time, where there exists an increasing trend towards avoiding axillary dissection at all in the presence of very small breast cancers with clinically negative axillae.
